# New species of *Habronattus* and *Pellenes* jumping spiders (Araneae, Salticidae, Harmochirina)

**DOI:** 10.3897/zookeys.646.10787

**Published:** 2017-01-17

**Authors:** Wayne P. Maddison

**Affiliations:** 1 Beaty Biodiversity Museum and Departments of Zoology and Botany, University of British Columbia, 6270 University Boulevard, Vancouver, British Columbia, V6T 1Z4, Canada

**Keywords:** Araneae, Salticidae, Plexippini, Harmochirina, jumping spider

## Abstract

The harmochirine jumping spiders include the New World *Habronattus*, notable for their complex courtship displays, and *Pellenes*, found throughout the Old World and North America. Five new species of *Habronattus* and one new species of *Pellenes* are here described from North America: *Habronattus aestus*, **sp. n.**, *Habronattus chamela* **sp. n.**, *Habronattus empyrus* **sp. n.**, *Habronattus luminosus* **sp. n.**, *Habronattus roberti* **sp. n.**, and *Pellenes canadensis* **sp. n.** For each of the new species, photographs of living specimens are given, as well as notes on habitat. The new subgenus Pellenattus is described for the subgroup of *Pellenes* restricted to North America, with type species *Pellenes peninsularis* Emerton, 1925. Species placed in Pellenes (Pellenattus) are *Pellenes apacheus* Lowrie & Gertsch, 1955, *Pellenes canadensis* **sp. n.**, *Pellenes crandalli* Lowrie & Gertsch, 1955, *Pellenes dorsalis* (Banks, 1898b), *Pellenes grammaticus* Chamberlin 1925, *Pellenes levii* Lowrie & Gertsch, 1955, *Pellenes limatus* Peckham & Peckham, 1901, *Pellenes longimanus* Emerton, 1913, *Pellenes peninsularis* Emerton, 1925, *Pellenes shoshonensis* Gertsch, 1934, and *Pellenes washonus* Lowrie & Gertsch, 1955. *Pellenes wrighti* Lowrie & Gertsch, 1955 is synonymized with *Pellenes peninsularis*. Attention is drawn to an undescribed species of *Habronattus* from Canada whose only known specimen is apparently lost.

## Introduction

The two jumping spider genera *Habronattus* F.O. Pickard-Cambridge, 1901 and *Pellenes* Simon, 1876 are closely related within the subtribe Harmochirina (Maddison and Hedin 2003a, b; Maddison 2015). While *Habronattus* species are confined to the Americas (Griswold 1987) and known for their complex courtship ornamentation and behaviour (Peckham and Peckham 1890; Griswold 1987; Elias et al. 2003, 2012), *Pellenes* are distributed throughout the Old World, along with North America, and show considerably less sexual dimorphism and courtship complexity. The phylogeny of *Habronattus* has been studied by both morphological (Griswold 1987) and molecular data (Maddison and Hedin 2003b), but many ambiguities remain — thus, an ongoing phylogenetic study seeks to use genomic data. To offer names for 6 taxa used in that phylogenomic study, five new species of *Habronattus* and one new species of *Pellenes* are described from North America. In addition, a new subgenus is erected to house the North American group of *Pellenes*.

## Methods

Specimens are deposited in the Spencer Entomological Museum of the University of British Columbia
(UBC-SEM), the Colección Nacional de Arácnidos, Instituto de Biología, Universidad Nacional Autónoma de México
(CNAN-IBUNAM), the Museum of Comparative Zoology, Harvard University (MCZ), or the American Museum of Natural History
(AMNH).

Preserved specimens were examined under both dissecting microscopes and a compound microscope with reflected light. Drawings (except that of *Pellenes peninsularis* habitus and palpi from Ontario) were made with a drawing tube on a Nikon ME600L compound microscope.

Terminology is standard for Araneae. The descriptions were written with primary reference to the focal specimen indicated, which was used for measurements and carefully checked for details, but they apply as far as known to the other specimens examined. All measurements are given in millimeters. Carapace length was measured from the base of the anterior median eyes not including the lenses to the rear margin of the carapace medially; abdomen length to the end of the anal tubercle. Rotation of the bulb of the palp expressed in degrees counterclockwise from distal. Thus, 0° is distal (12:00 on an analog clock face); 90° is prolateral (9:00); 180° is proximal (6:00); 270° is retrolateral (3:00). The following abbreviations are used: AME, anterior median eyes; ALE, anterior lateral eyes; PLE, posterior lateral eyes; PME, posterior median eyes (the “small eyes”); RTA, retrolateral tibial apophysis. The apophysis accompanying the embolus of the male palp was called the conductor by Lowrie and Gertsch (1955), the tegular apophysis by Griswold (1987), and the compound terminal apophysis by Logunov et al. (1999). It is here called the “terminal apophysis”, abbreviated “TmA”, following Edwards (2015).

## Taxonomy

### Genus Habronattus

Taxon classificationAnimaliaAraneaeSalticidae

F. O. Pickard-Cambridge, 1901

#### Type species.

*Habrocestum mexicanum* Peckham & Peckham, 1896

#### Notes.

Most of the approximately 100 species (Griswold, 1987) of *Habronattus* are found in Mexico and the United States, extending into arctic Canada and to southern South America. *Habronattus* as a whole is easily recognized by the 90° bend (“elbow”) on the long thin TmA of the palp, though the elbow is lost secondarily in the *Habronattus coecatus* species group. Several clades of species are recognized as species groups (Griswold 1987; Maddison and Hedin 2003b), some referred to here in the descriptions.

Two of the new species were studied by Maddison and Hedin (2003b): *Habronattus chamela*, called “*Habronattus* sp. (CHMLA)” by Maddison & Hedin, and *Habronattus roberti*, called “*Habronattus* sp. (ROBRT)”. For the other new species we used informal names in field and lab notebooks: *Habronattus aestus* as “peñasco” or “ESTU”, *Habronattus empyrus* as “blondie” or “BLNDI”, *Habronattus luminosus* as “sunglow” or “SUNGL”, and *Pellenes canadensis* as “*Pellenes* cf. *levii*”. Images and mentions of *Habronattus luminosus* have appeared on news reports in connection with Zurek et al.’s (2015) study of colour vision, under the name “*Habronattus sunglow*”.

### Habronattus aestus

Taxon classificationAnimaliaAraneaeSalticidae

Maddison sp. n.

http://zoobank.org/43D988D1-AA30-45F6-8A90-15D67B59EAA7

Figs 1–12

#### Holotype.

Male in CNAN-IBUNAM, with data: México: Sonora: Puerto Peñasco, Estero Morúa, 31.30°N 113.46-113.48°W, 22–23 February 2003, W. Maddison, WPM#03-001.

#### Paratypes.

(3 ♂♂ 3♀♀): Same data as holotype (1♂ in UBC-SEM, 1♂ in AMNH). México: Sonora: Puerto Peñasco, Estero Cerro Prieto, 31.418°N 113.626°W, 1 m elev., 18 August 2013, W. Maddison & A. Meza López, WPM#13-086 (1♂ [specimen AZS13-7854, Figs 5–7] 2♀♀ in UBC-SEM, 1 ♀ in AMNH).

**Figures 1–12.**
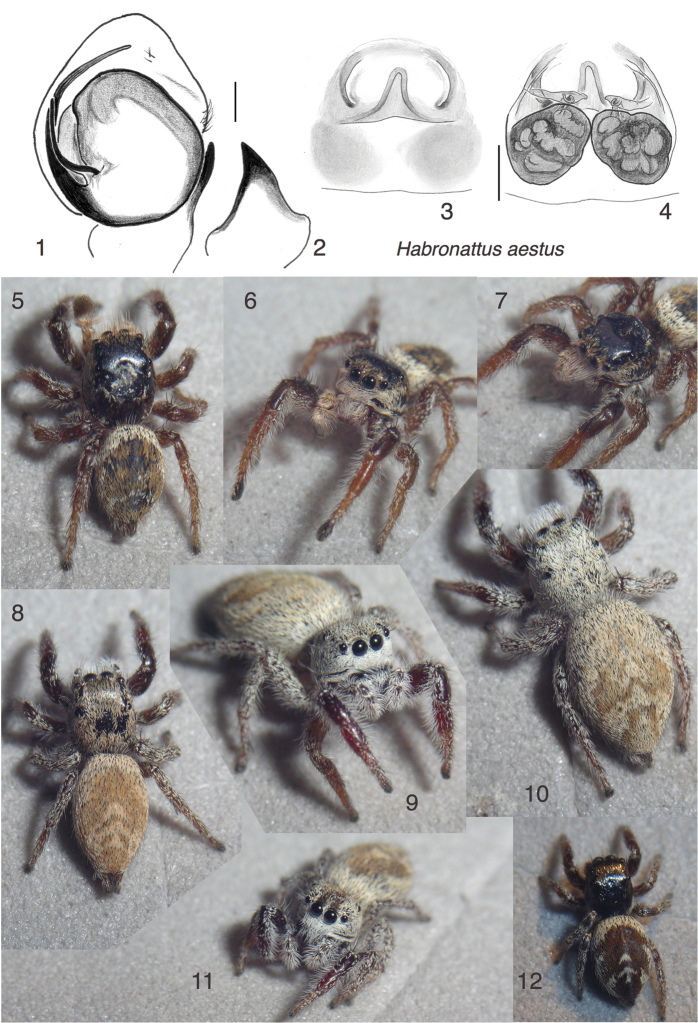
*Habronattus aestus* sp. n. **1** Left male palp, ventral view (holotype) **2** Same, retrolateral view of palp tibia **3** Epigynum, ventral view (paratype described) **4** Epigynum, cleared, dorsal view, same female **5–7** Male AZS13-7854 (paratype) **8** Female AZS13-7889 **9–10** Female AZS13-7885 **11** Female AZS13-7874 **12** Juvenile AZS13-8283. All specimens are from Estero Cerro Prieto (WPM#13-086) except holotype, from Estero Morúa (WPM#03-001). Figures 5–12 are copyright © 2015 W. P. Maddison, released under a Creative Commons Attribution (CC-BY) 3.0 license.

#### Etymology.

From the Latin, in reference to the tides and the heat of its habitat.

#### Diagnosis.

This species can be placed in the *americanus* group by male ornamentation (shelf of projecting setae under the AME, Fig. 7; darkened first tarsus, Fig. 6) and the relatively short and pointed terminal apophysis (TmA) arising at about 120°. However, unlike other members of the group (Griswold 1987, figures 184–186), the TmA of *Habronattus aestus* is thin at the base (Fig. 1), more or less lacking the elbow typical of *Habronattus*. The male’s scantiness of ornamentation is also distinctive — the first males found were not recognized as adult initially — as is the habitat of saline negative estuaries. Both male and female have the first tibia reddish brown, contrasting against a darker patella (Figs 6 and 9).

#### Description.

*Male* (focal specimen: holotype). Carapace length 1.9; abdomen length 1.8. Palp (Figs 1–2) with bulb little rotated, embolus arising at about 140°; TmA thin and with only a hint of an elbow. RTA triangular. First leg with tarsus and metatarsus thicker than usual. Colour: Chelicerae pale, covered with erect white setae. Palp femur, patella, tibia with partially erect white setae, especially long prolaterally. Distal 3/4 of cymbium with fine dark hairs. Legs light to medium brown in alcohol, though darker in life. First leg metatarsus and tarsus dark, with extended dark scopula. Clypeus covered with cream coloured scales, with prominent row of long cream-coloured setae extending forward, forming a shelf (Fig. 7). Carapace dark brown with bronze scales. Abdomen similar to that of the juvenile in Fig. 12, reddish brown with broad paler basal band, two distinctive cream triangles centrally, and lateral cream bands made of paired crescents.

*Female* (focal specimen: paratype, specimen from Estero Cerro Prieto, Figs 3–4). Carapace length 2.3; abdomen length 3.0. Epigynum with semicircular atria; central pocket broad posteriorly (Fig. 3). Colour: Chelicerae medium brown. Legs medium to pale brown, the first pair darkest. Clypeus covered with white scales, with (as in male) shelf of long white setae projecting forward (Fig. 10). Carapace and abdomen covered with cream-coloured scales except for orange-tan patches on abdomen. Central pale triangles (chevrons) on dorsum connected to lateral bands, as in Figs 8 and 10.

#### Additional material examined.

Two juveniles and 3 females from the type locality.

#### Natural history.

Found only in the negative tidal estuaries of Sonora, México. These unusual habitats have salt-tolerant plants (such as *Salicornia*) on soil that is constantly wet with salt water, as the tides enter then drain to cut stream-like channels (Figs 95–96). Fresh water is rarely available in this harsh desert. Although *Habronattus aestus* was found at Estero Morúa, in 2013 it was considerably more common at Estero Cerro Prieta. There, it was found either in retreats in the larger salt-tolerant plants along the edges of the channels, about 20-40 cm above the substrate, or on the mud/sand of the slopes of these channels after shaking the overhanging salt-tolerant plants. The courtship involves behaviours similar to those seen in other *americanus*-group members, though of weak amplitude. A video of the courtship of male AZS13-7854 is available at https://youtu.be/JUkULLdOZ0w.

### Habronattus chamela

Taxon classificationAnimaliaAraneaeSalticidae

Maddison sp. n.

http://zoobank.org/65A538DC-EAEC-482F-978D-29CF544AA9D8

Figs 13–25

#### Holotype.

Male specimen JAL14-9837 in CNAN-IBUNAM, with data: México: Jalisco: Estación de Biología Chamela, 400-650 m on Calandria Trail, 19.5038 - 19.5045°N 105.0334 - 105.0344°W, 19 Feb. 2014, W. Maddison & H. Proctor WPM#14-034.

#### Paratypes

(5♂♂ 7♀♀). Same data as holotype (1♀ specimen JAL14-9844 in CNAN-IBUNAM. 1♀ specimen JAL14-9840 in UBC-SEM, 1♀ in AMNH). México: Jalisco: Estación de Biología Chamela, 19.498° N 105.045° W, 1-2 June 1998, W. Maddison et al, WPM#98-071 (1♂ in AMNH, 1♂ in MCZ, 2♂♂ 2♀♀ in UBC-SEM). México: Jalisco: Estación de Biología Chamela 400-850 m on Calandria Trail, 19.5023-19.5045°N 105.0328-105.0344°W, 19 Feb. 2014, W. Maddison & H. Proctor, WPM#14-033 (1♀ specimen JAL14-9847 in UBC-SEM). México: Jalisco: Estación de Biología Chamela, Calandria Trail, 19.501 - 19.505°N 105.035°W, 130 m elev., 23 Feb. 2014, W. Maddison & R. Sosa, WPM#14-038 (1♂ specimen JAL14-0138 Fig. 19 in UBC-SEM, 1♀ in MCZ).

**Figures 13–25.**
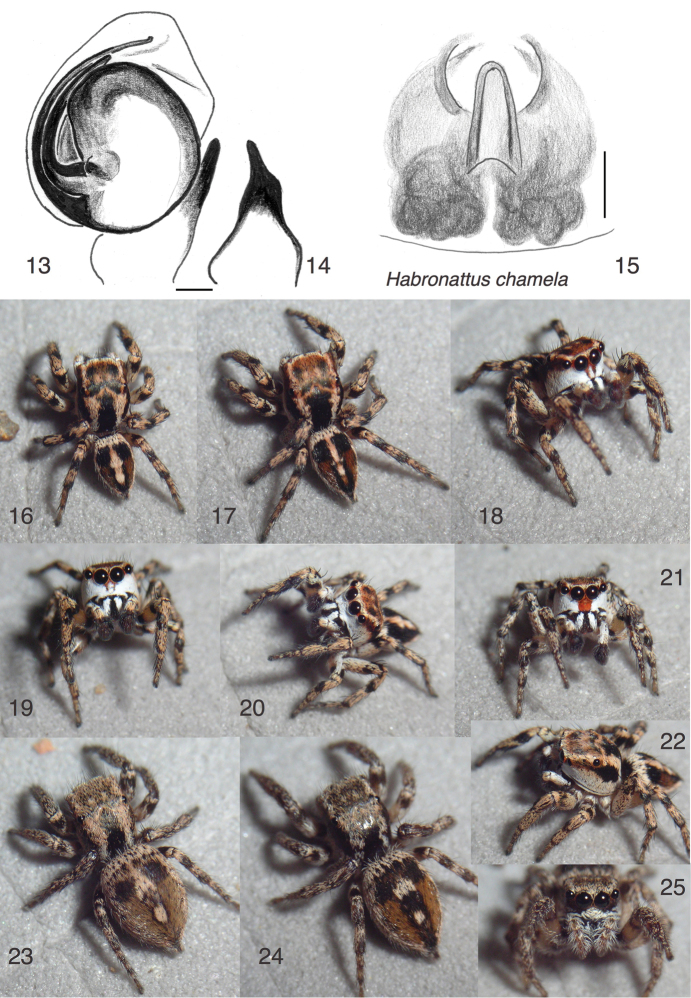
*Habronattus chamela* sp. n. **13** Left male palp, ventral view (paratype male) **14** Same, retrolateral view of palp tibia **15** Epigynum, ventral view (paratype female) **16** Male JAL14-9795 **17–18** male JAL14-9812 **19** Male JAL14-0138 (paratype) **20** male JAL14-0224 **21** male JAL14-0587 **22** male JAL14-0213 **23** female JAL14-9844 (paratype) **24–25** female JAL14-8947. All specimens are from Estación de Biología Chamela or Chamela Estuary except Fig. 21, from El Tuito (WPM#14-047). Figures 16–25 are copyright © 2015 W. P. Maddison, released under a Creative Commons Attribution (CC-BY) 3.0 license.

#### Etymology.

The name of the type locality is placed as a noun in apposition.

#### Diagnosis.

This little-ornamented species appears to be close to *Habronattus nahuatlanus* Griswold 1987. The male’s white clypeus is divided by one or two central dark bands beneath and between the AME (Figs 18, 19, 21), separating it from most other *Habronattus* except *Habronattus nahuatlanus* and *Habronattus banksi* (Peckham & Peckham, 1901), from which it differs in having a much less rotated bulb of the palp. In some specimens of *Habronattus chamela*, the dividing bands are absent (Fig. 20). The bulb of the palp is unusually little rotated (Fig. 13), with the base of the TmA pointing prolaterally (to 90°) as in *Habronattus paratus*, *Habronattus moratus*,﻿ and the *americanus* group, from which *Habronattus chamela* differs in many aspects of markings and form.

#### Description.

*Male* (focal specimen: holotype). Carapace length 2.0; abdomen length 1.8. Palp with bulb little rotated, with embolus arising at 150° and the base of TmA directed prolaterally (Fig. 13). RTA long with fingerlike projection (Fig. 14). Colour (Figs 16–21): Chelicerae dark with a patch of white scales (Figs 18–21). Palp femur and patella pale yellow, contrasting against dark tibia and cymbium. Femora of legs pale centrally, with black annulae proximally and distally. Other segments medium brown (with cream scales) with black annulae distally. Prolateral side of first tibia and metatarsus black. Clypeus covered with white scales except for two vertical black lines near the midline. Extending from clypeus is a broad marginal band of white scales, reaching to the back of the carapace where it contacts the narrow longitudinal bands descending from just inside the PME. Carapace otherwise mostly black or dark brown, except for faint inverted “V” between PME and two small spots in the middle of the ocular area (Fig. 17). Abdomen dark above with a cream sword-shaped longitudinal band along the midline, and with lateral cream lines. The dark areas are black in the anterior third, but reddish in the posterior two-thirds.

*Female* (focal specimen: paratype, specimen JAL14-9844, Fig. 23). Carapace length 2.1; abdomen length 2.7. Epigynum with central pocket long (Fig. 15); atria separate, not joined anteriorly. Colour: Chelicerae dark with a few white scales on basal half. Legs medium brown but with distinctly paler area centrally on femora. Clypeus dark except for white scales along the margin, extending upward at the midline. Carapace and abdomen as in the male but with lower contrast. The central longitudinal band of the abdominal dorsum is usually broken into two cream-coloured spots.

#### Geographical variation.

Males from the area of El Tuito, north of the type locality, have a continuous red patch in the centre of the clypeus (Fig. 21), instead of two vertical lines.

#### Additional material examined.

12♂♂ 1♀ in UBC-SEM: México: Jalisco: El Tuito, Rancho Primavera, 20.3447°N 105.3537°W, 700 m elev., 3 March 2014, W. Maddison, WPM#14-047 (4♂♂); México: Jalisco: Sierra Manantlan, 19.7013°N 104.3918°W, 1550 m elev., 1 June 1998, W. Maddison et al., WPM#98-067 (7♂♂ 1♀). México: Jalisco: Estación de Biología Chamela, 19.498°N 105.045°W, 1-2 June 1998, W. Maddison et al., WPM#98-071 (1♂).

#### Natural history.

Known from the tropical deciduous forests along the southern coast of Jalisco, México (Fig. 101), typically found on leaf litter or sticks on the ground that receives sun but is somewhat shaded (Fig. 99) — in contrast to the more open sunny ground on which *Habronattus roberti* lives nearby. The courtship involves the male standing at a distance from the female with first legs spread; he walks in bursts toward the female, sidling somewhat. On each burst forward, the front legs are flicked upward and the palps lowered to expose the face. A video of male courtship is available at https://youtu.be/mgXhB61u0mA.

### Habronattus empyrus

Taxon classificationAnimaliaAraneaeSalticidae

Maddison sp. n.

http://zoobank.org/1FCF2BA2-B4E8-4C5C-B384-7198F62E5D17

Figs 26–37

#### Holotype.

Male in CNAN-IBUNAM, with data: México: Sonora: Puerto Peñasco, Estero Morúa, 31.293 - 31.295°N 113.456 - 113.459°W, 17 August 2013, Maddison/Proctor/Evans/Leduc-Robert/Meza, WPM#13-084.

#### Paratypes

(5♂♂ 4♀♀). Same data as holotype (1♀ specimen AZS13-7828 in CNAN-IBUNAM, 1♂ 1♀ in UBC-SEM, 1♂ 1♀ in MCZ, 1♂ 1♀ in AMNH). México: Sonora: Puerto Peñasco, Estero Morúa, 31.293 - 31.294°N 113.456 - 113.458°W, 1 m elev., 16 August 2013, Maddison/Proctor/Evans/Leduc-Robert, WPM#13-079 (2♂♂ in UBC-SEM specimens AZS13-7562 [Fig. 30] and AZS13-7582 [Fig. 31]).

**Figures 26–37.**
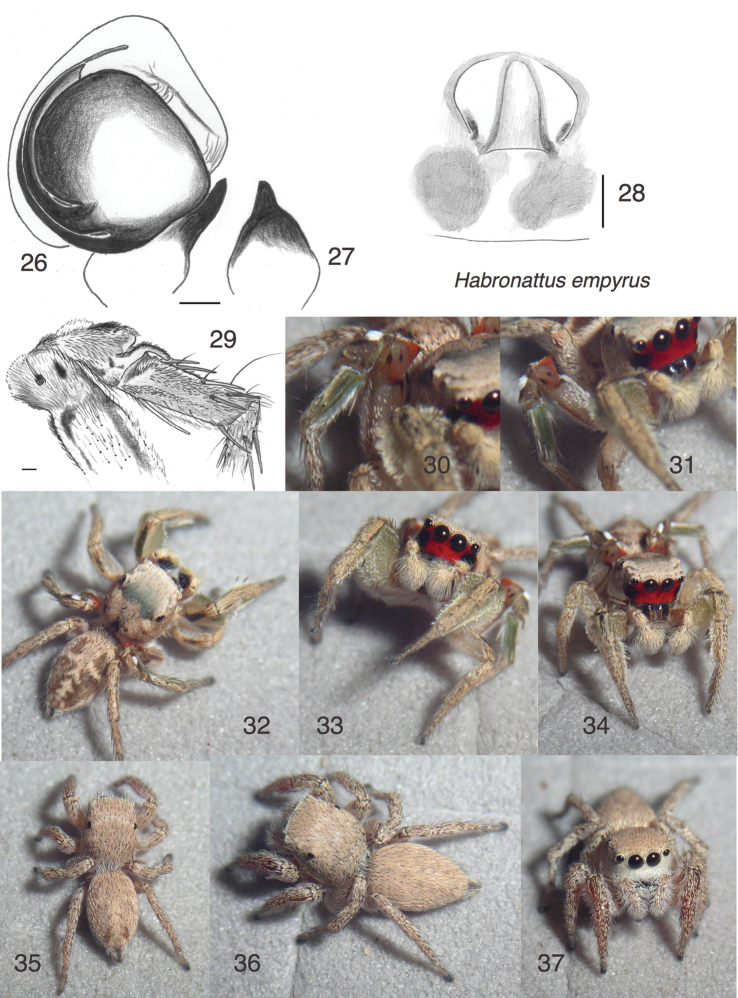
*Habronattus empyrus* sp. n. **26** Left male palp, ventral view (holotype male) **27** Same, retrolateral view of palp tibia **28** Epigynum, ventral view (paratype female AZS13-7828) **29** Femur, patella and tibia of third leg, prolateral view (holotype male) **30** Male AZS13-7562 (paratype) **31** Male AZS13-7582 (paratype) **32–33** Male AZS13-7575 **34** Male AZS13-7834 **35** Female AZS13-7617 **36–37** Female AZS13-7828 (paratype). All specimens are from Estero Morúa (WPM#13-084) except Figs 30–33, 35 from Estero Cerro Prieto (WPM#13-079). Figs 30–37 are copyright © 2015 W. P. Maddison, released under a Creative Commons Attribution (CC-BY) 3.0 license.

#### Etymology.

From the Greek *empyros*, burning, referring to the male’s flaming colors: a brilliant red face against a pale yellow-orange body and legs. Also, to the author’s synesthesia, the dominant letters of the name match the colours of males perfectly: “e” for the green first legs, “r” for the red face, and “y” and “s” for the pale yellow-orange of the body and legs.

#### Diagnosis.

A member of the *coecatus* group distinctive for its pale colours. The male’s red face and form of the third legs (Figs 29–31, 33, 34) distinguish it from all other members of the *coecatus* group except *Habronattus pyrrithrix*, from which it differs in having a much paler body, the green first legs paler in life, and the red facial band narrower. The third leg is much like that of *Habronattus pyrrithrix*, *Habronattus carpus* and *Habronattus mexicanus*, with an orange tuft on the dorsal distal side of the femur and a dark patella with pale speckles, a bright white dorsal-basal tuft, and a moderate but thin thumb-like dorsal-distal apophysis (Figs 29–31; compare to Griswold 1987 figures 84–85). However, the femur of *Habronattus empyrus* has an additional black streak just ventral to the prominent macroseta on the prolateral distal face of the femur (Fig. 29). Typical *Habronattus pyrrithrix* were found only 6 km away from the type locality, lessening concerns that *Habronattus empyrus* might be a only a geographical variant.

#### Description.

*Male* (focal specimen: holotype). Carapace length 2.2; abdomen length 2.1. Palp typical for *coecatus* group, with sickle-shaped TmA. Embolus arises at 180° (Fig. 26). Colour and ornaments in alcohol: Chelicerae dark at base, paler at tips. Palp femur and tibia pale except dark patch prolaterally and ventrally. Cymbium pale yellow-brown with long white hairs. Legs pale yellowish except for dorsal black stripe on first femur and markings of third leg. First leg with fringes and modified spatulate setae typical of *coecatus* group. Third femur with longitudinal black lines on dorsal and ventral edges of prolateral face, up to the expanded distal area which bears two black spots and a dorsal tuft of orange setae (Figs 29, 30). Third patella with a typical expanded triangular ridge above and a thumb-like apophysis distally. Clypeus red, transitioning abruptly to black between the AME and ALE. The black is a fairly narrow region beneath the ALE. Otherwise, the carapace is covered fairly uniformly with cream to light yellowish-brown scales, with the usual *coecatus*-group markings indistinct. Abdomen with standard *coecatus*-group markings of a central pale chevroned longitudinal band with a transverse band cutting across it, but less distinct than usual, because the background is light brown rather than black. In life (Figs 30–34), the palp femur is light brown, not red as in *Habronattus pyrrithrix*. The integument of the first leg is light green. The third tibia is also green.

*Female* (focal specimen: paratype, specimen AZS13-7828; Figs 28, 36–37). Carapace length 2.6; abdomen length 2.7. Structure (including epigynum, Fig. 28) typical for *coecatus* group. Colour (Figs 35–37) typical for *coecatus* group, pale beige to light brown. Clypeus white (Fig. 37). Abdomen shows only a trace of the markings of the male.

#### Additional material examined.

10♂♂ 7♀♀ in UBC-SEM: México: Sonora: Puerto Peñasco, Estero Morúa, 31.293°N 113.452°W, 1 m elev., 16 August 2013, S.C. Evans, WPM#13-078 (1♂). México: Sonora: Puerto Peñasco, Estero Morúa, 31.293 - 31.294°N 113.456 - 113.458°W, 1 m elev., 16 August 2013, Maddison/Proctor/Evans/Leduc-Robert, WPM#13-079 (3♂♂). México: Sonora: Puerto Peñasco, Estero Morúa, 31.293 - 31.295°N 113.456 - 113.459°W, 17 August 2013, Maddison/ Proctor/Evans/Leduc-Robert/Meza, WPM#13-084 (3♂♂ 7♀♀). México: Sonora: Puerto Peñasco, Estero Morúa, 31.296 - 31.297°N 113.487 - 113.493°W, 17 August 2013, Maddison/ Proctor/Evans/Leduc-Robert, WPM#13-085 (2♂♂). México: Sonora: Puerto Peñasco, Estero Cerro Prieto, 31.418°N 113.626°W, 1 m elev., 18 August 2013, W. Maddison & A. Meza López, WPM#13-086 (1♂).

#### Natural history.

Found with *Habronattus aestus* in the negative tidal estuaries of Sonora, México. *Habronattus empyrus*, however, was found in the flatter areas with short salt-tolerant plants including *Salicornia* (Fig. 97), unlike *Habronattus aestus* which was associated with large salt-tolerant plants along the tidal channels. Individuals of *Habronattus empyrus* were found on the wet sand/mud, or hopping from one short plant to another like little monkeys. A portion of courtship was observed, and appears typical for the *Habronattus coecatus* species group (https://youtu.be/Lwa678NVC3U).

### Habronattus luminosus

Taxon classificationAnimaliaAraneaeSalticidae

Maddison sp. n.

http://zoobank.org/DB648F11-9FE1-41A9-9FBB-A6FA1E8D2352

Figs 38–49

#### Holotype.

Male in UBC-SEM, with data: U.S.A.: Arizona: Santa Cruz Co., Mt. Hopkins Road, Amateur Astronomy Vista, 31.6775°N 110.9288°W, 7 May 2014, N. Morehouse & D. Zurek.

#### Paratypes

(1♂ 2♀♀). U.S.A.: Arizona: Santa Cruz Co., Mt. Hopkins Rd, Amateur Astronomy Vista, 31.6759 - 31.6762°N 110.9289 - 110.9293°W, 1430 m elev., 7 August 2013, W. Maddison & H. Proctor, WPM#13-056 (2♀♀ specimens ASZ13-7108 and AZS13-7081 in UBC-SEM). Arizona: Cochise Co., Sunglow, west side Chiricahua Mts., 12 July 1977, B. & V. Roth (1♂ in AMNH).

#### Etymology.

Latin, “full of light”, in reference to the pale coloring of the body, especially in the yellow-white juveniles, as well as to the name of the locality of the first known specimen, Sunglow, Arizona.

#### Diagnosis.

**Figures 38–49.**
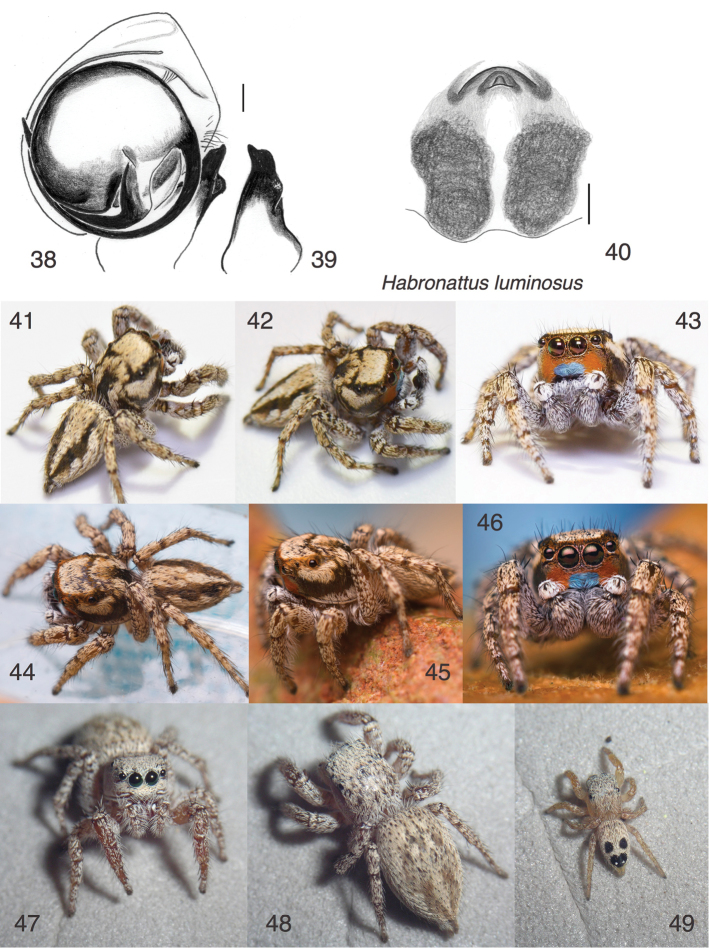
*Habronattus luminosus* sp. n. **38** Left male palp, ventral view (paratype male from Sunglow) **39** Same, retrolateral view of palp tibia **40** Epigynum, ventral view (paratype female AZS13-7108 from Amateur Astronomy Vista WPM#13-056) **41–43** Male holotype (photographed by Daniel Zurek) **44–46** Male from Mt. Hopkins Road 31.6697°N 110.9147°W (photographed by Thomas Shahan) **47, 48** Female paratype AZS13-7108 from Amateur Astronomy Vista WPM#13-056 **49** Juvenile AZS13-6951 from Mt. Hopkins Road WPM#13-054. Figs 41–43 are ©2016 Daniel Zurek, released under a Creative Commons Attribution (CC-BY) 4.0 license. Figs 44–46 are ©2016 Thomas Shahan, released under a Creative Commons Attribution-NonCommercial-ShareAlike 3.0 Unported license. Figs 47–49 are copyright © 2015 W. P. Maddison, released under a Creative Commons Attribution (CC-BY) 3.0 license.

A large-bodied species, covered extensively with pale scales in both males and females. The male is distinctive for the red face with a blue central patch (Figs 43, 46), though in alcohol the blue patch appears as metallic green. The tibial apophysis of the palp has a distinct bump projecting retrolaterally, proximal from the tip (Figs 38, 39). The epigynum is distinctive, with the central pocket for the RTA very small, on a mound in front of which the openings are adpressed (Fig. 40).

#### Description.

*Male* (focal specimen: holotype, Figs 41–43). Carapace length 3.1; abdomen length 3.2. Palp (Figs 38–39): bulb moderately rotated, with embolus arising at about 290°. RTA with a notable bump dorsally near the tip. Legs: unornamented, as in the female. Carapace: At the back of the carapace is a small stridulatory file, as is seen in various *Habronattus* species (Maddison and Stratton 1988). The file is similar in form to that of the *Habronattus agilis* species group, but much narrower. Colour in alcohol: Chelicerae brown with fine glistening hairs. Palp femur light brown with some white scales; tibia with white scales; cymbium brown with a few pale setae. Legs without distinct markings, light to medium brown, with pale scales. Clypeus brown, with a central patch just over the chelicerae of metallic green setae — this contrasts with the appearance in life of a rust-coloured clypeus with a central blue patch (Figs 43, 46). Carapace dark brown with patches of cream-coloured scales in the ocular area, below the PLE, and on the thorax (Figs 41–42, 44). Abdomen dark brown above with a longitudinal band of cream scales medially, wider at front. Sides also covered in cream scales. Venter medium gray-brown.

*Female* (focal specimen: paratype, specimen AZS13-7108, Figs 47–48). This is the most intact female, as specimen AZS13-7081 (= genetic voucher GLR218) was mostly consumed for RNA extraction. However, an acrocerid fly from ASZ13-7108 emerged about 7 weeks after capture, and thus the abdomen is collapsed. While acrocerid parasitism can affect development, the epigynum of the parasitized specimen (Fig. 40) is apparently natural, as it matches closely to that of AZS13-7081. Carapace length 3.1. Epigynum (Fig. 40) with very small central pocket (the guide for the RTA) embedded within a sclerotized mound. Openings just in front of this mound, almost hidden by it. Colour: Markings indistinct; appendages and body pale yellowish to medium brown, darkest in the ocular area, covered with cream-coloured scales. Abdomen with hint of markings of male (Fig. 48). Clypeus (Fig. 47) covered in cream scales.

#### Additional material examined.

1♂, 8 juveniles, all from southeastern Arizona. U.S.A.: Arizona: Santa Cruz Co., Mt. Hopkins Rd, Amateur Astronomy Vista, 31.6759 - 31.6762°N 110.9289 - 110.9293°W, 1430 m elev., 7 August 2013, W.Maddison & H. Proctor, WPM#13-056 (2 juveniles in UBC-SEM). U.S.A.: Arizona: Santa Cruz Co.: Mt. Hopkins Road, 31.6705°N 110.9137°W, 1640 m elev., 6 August 2013, W.Maddison & H. Proctor, WPM#13-054 (3 juveniles in UBC-SEM). Arizona: Pima Co.: Madera Canyon, near Proctor Road, 31.7417°N 110.8847°W, 9 August 2013, W. Maddison, WPM#13-062 (3 juveniles in UBC-SEM). Arizona: Santa Cruz Co., Mt. Hopkins Road, 31.6697°N 110.9147°W, 19 June 2012, M. Girard (1♂ in UBC-SEM, DNA voucher d436 and Figs 44–46).

#### Natural history.

After this species was first found by Barbara and Vince Roth in the Chiricahua Mountains in 1977, it went uncollected for many years, despite my many attempts to find it in southern Arizona when I resided there for 13 years. It was then rediscovered in 2012 by Madeline Girard in the high desert scrub/grassland just below the oak woodlands in the Santa Rita Mountains. Subsequent collecting has revealed an unusual habitat: it is found hidden in tall clumps of grass in the desert scrub near the lower edge of the oak woodlands (Figs 102–103). It can be found by lifting up the overhanging grass of the clump, or pushing apart the clump to reveal specimens hidden near its core. Although we did not find many adults in August, small yellowish juveniles with prominent black spots (Fig. 49) were reasonably common. A few were raised for several moults, and by their change in markings, they do appear to be *Habronattus luminosus*, supported also by the fact that they cannot be assigned to any other known *Habronattus* in the relatively well-known fauna of southern Arizona. Given the known localities and grassy habitat, its range might be expected to extend into the Chihuahan Desert grasslands.

### Habronattus roberti

Taxon classificationAnimaliaAraneaeSalticidae

Maddison sp. n.

http://zoobank.org/A92F955E-13A9-4EDE-A707-F49C6253041C

Figs 50–62 , 63–68

#### Holotype.

Male specimen JAL14-0175 in CNAN-IBUNAM, with data: México: Jalisco: Estación de Biología Chamela, Calandria Trail, 19.501 - 19.505°N 105.035°W, 130 m elev., 23 Feb. 2014, W. Maddison & R. Sosa, WPM#14-038.

#### Paratypes

**Figures 50–62.**
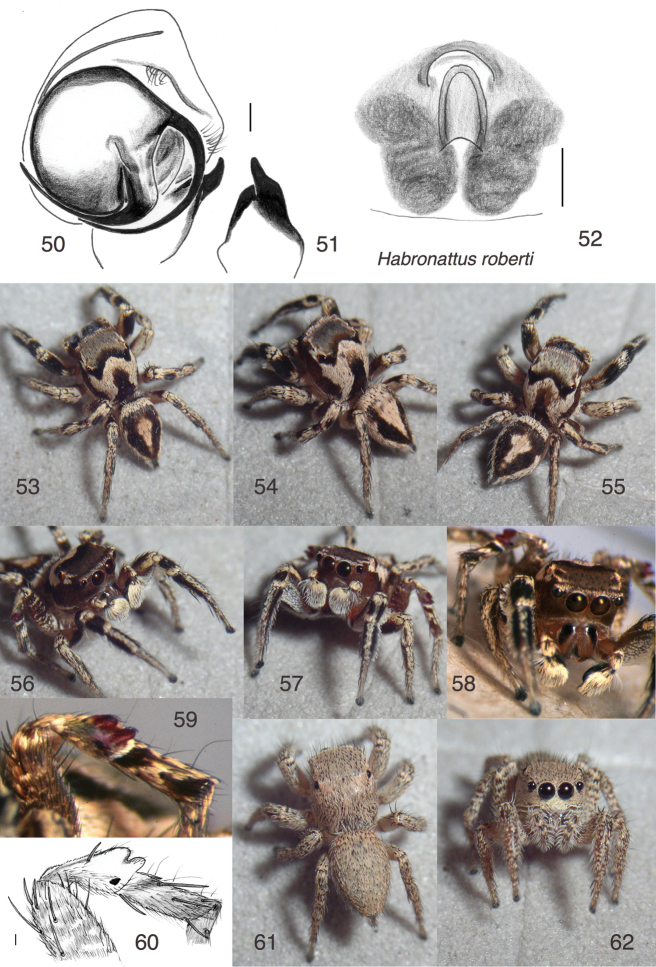
*Habronattus roberti* sp. n., typical (coastal) populations. **50** Left male palp, ventral view (paratype male W257 from WPM#98-071) **51** Same, retrolateral view of palp tibia **52** Epigynum, ventral view (paratype female from WPM#98-070) **53, 56** Male JAL14-8934 from WPM#14-015 **54, 57** Male JAL14-9737 from WPM#14-034 **55** Male JAL14-9777 from WPM#14-034 **58** Male from WPM#98-070 **59** Femur, patella and tibia of third leg, prolateral view, of male from WPM#98-070 **60** Same, in alcohol, of male paratype #257 from WPM#98-071 **61–62** Female paratype JAL14-9239 from WPM#14-015 All specimens are from the area of Chamela, Jalisco; WPM collecting codes are those indicated in specimen records in description. Figs 53–59, 61, 62 are copyright © 2015 W. P. Maddison, released under a Creative Commons Attribution (CC-BY) 3.0 license.

(5♂♂ 7♀♀). Same data as holotype (2♂♂ specimens JAL14-0184 and JAL14-0152 in UBC-SEM). México: Jalisco: Chamela estuary, 19.5290°N 105.0770°W, 2 June 1998, W. Maddison et al., WPM#98-070 (1♀ Fig. 52 in UBC SEM, 1♀ in AMNH). México: Jalisco: Estación de Biología Chamela, 19.498°N 105.045°W, 1-2 June 1998, W. Maddison et al., WPM#98-071 (1♂ specimen W257 Figs 50–51 in UBC SEM). México: Jalisco: Estación de Biología Chamela, Chachalaca Trail, 19.496°N 105.042°W 10 Feb. 2014, W. Maddison & H. Proctor, WPM#14-015 (1♂ specimen JAL14-9252 and 1♀ specimen JAL14-9239 in UBC-SEM). México: Jalisco: Estación de Biología Chamela, Viveros, 19.499°N 105.043°W, 90 m elev., 16 - 27 Feb. 2014, W. Maddison & H. Proctor, WPM#14-028 (1♀ specimen JAL14-0120 in UBC-SEM, 1♂ in AMNH). México: Jalisco: Estación de Biología Chamela, Viveros, 19.499°N 105.043°W, 90 m elev., 28 Feb. - 1 March 2014, W. Maddison, WPM#14-042 (1♀ in CNAN-IBUNAM, 1♀ in MCZ, 1♀ in MCZ).

#### Etymology.

Named after my late father, Robert John Maddison, who introduced me to the small things in nature through fishing bait and saturniid cocoons. When my brother and I developed interests in beetles and spiders, he offered to take the family on long collecting trips. His gentle encouragement let me find my own love for the riches of biodiversity.

#### Diagnosis.

Belonging within the clade whose males have modified first and third legs (*coecatus*, *viridipes* and *clypeatus* groups), but not clearly belonging to any of the subgroups. Shows similarities to the *viridipes* species group (a ridge of raised scales between the PLE; courtship behaviour) but also to the *clypeatus* group (red-purple third patella; checkered or striped pattern visible in male AMEs). Unlike relatives with green legs, the yellow-green of the first leg of northern populations is weakly green and is restricted to the underside of the femur. Modifications of the third patella are small, in that respect resembling several species of the *viridipes* and *clypeatus* groups, but differing from those in having two small bumps dorsally on the patella (Figs 59–60, 67–68). Like *Habronattus moratus* (Gertsch & Mulaik, 1936), the raised ridge of scales between the PME appears from in front as a tuft over the PME (white arrow Fig. 66) and a broader raised ridge that is bimodal (lower at the midline; black arrow Fig. 66). Unlike *Habronattus moratus*, the palp’s bulb is reasonably well rotated (*Habronattus moratus*, TmA base pointing to 90°; *Habronattus roberti*, TmA base pointing to 190°).

**Figures 63–68.**
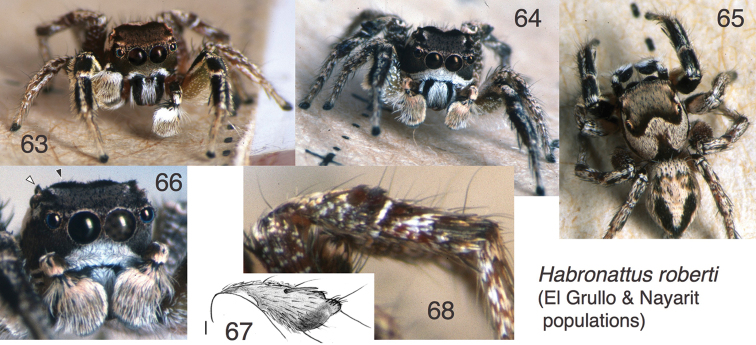
*Habronattus roberti* sp. n., El Grullo and Nayarit populations. **63, 65** Male from Jalisco: Los Yesos, near El Grullo 19.750°N 104.067°W WPM#98-065 **64, 66** Male from Nayarit: Singaita, just E of San Blas **67, 68** Femur, patella and tibia of third leg, prolateral view, of male from Los Yesos (WPM#98-065); Fig. 68 is of the right leg, digitally flipped horizontally. Figs 63–66, 68 are copyright © 2015 W. P. Maddison, released under a Creative Commons Attribution (CC-BY) 3.0 license.

#### Description.

**Figures 69–70.**
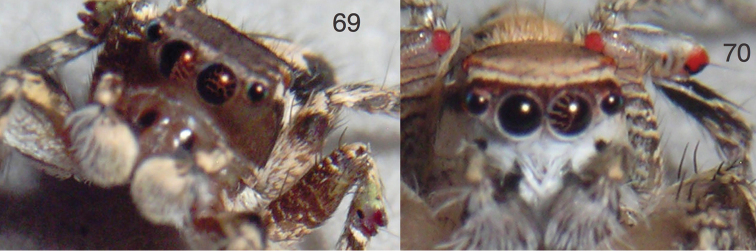
*Habronattus* males with checkered and striped patterns in the AME. **69** *Habronattus roberti* male JAL14-9777 from WPM#14-034 **70** *Habronattus aztecanus* male JAL14-8782 from Jalisco: Puerto Vallarta, Bocanegra beach, 20.670°N 105.274°W, 3 m elev., 8 Feb. 2014, WPM#14-012. Figures 69–70 are copyright © 2015 W. P. Maddison, released under a Creative Commons Attribution (CC-BY) 3.0 license.

*Male* (focal specimen: holotype). Carapace length 2.3; abdomen length 2.3. Palp’s bulb well rotated, with embolus arising at 310° (Fig. 50). Tip of RTA in ventral view curves toward the prolateral, forming a hook, like that seen in *Habronattus arcalorus* Maddison & Maddison, 2016 and other *clypeatus*-group members. In retrolateral view, the RTA is more robust than usually seen in the *viridipes* group. Colour: Chelicerae light brown with a glabrous black patch in basal prolateral portion (Fig. 58). Palp femur pale brown with white setae distally; patella covered with white setae; tibia darker but with long white setae retrolaterally; cymbium covered with white setae. First leg with long fringe of white setae on ventral-retrolateral edge, longest distally; weak fringe ventrally and retrolaterally on patella and tibia; no prolateral fringe. One prolateral macroseta on tibia longer than usual, and only very slightly flattened. First leg black above, brown otherwise, palest beneath the femur where it is covered with white setae expanded at the tip (Figs 57–58). (In life, the integument of the femur shows no obvious green.) Other legs medium to dark brown with cream coloured scales. Second leg with prolateral side distinctly darker. Third femur with faint transverse bands of cream scales as in *viridipes* and *clypeatus* group (Fig. 56). Patella with a black spot on the prolateral side, and two protuberances dorsally, red-purple in life (Figs 59–60). (In some specimens, the proximal half of the patella is yellowish-green, Fig. 69.) The prolateral face of the tibia has a narrow strongly white band proximally, then a black region, then a rising band of cream scales. Clypeus and sides of carapace brown. Ocular area dark, covered in grey-brown scales that grade to black between the PME. On the thorax, broad bands of cream scales extend from beneath PME and PLE to the posterior margin; these two bands are contiguous with the inverted V of cream scales between the PME. The integument underlying these thoracic bands is pale. Abdomen black to dark brown above with a broad cream basal band, and a central longitudinal band that is widest at front, sometimes contacting the basal band (Fig. 54). Venter with three longitudinal dark bands.

*Female* (focal specimen: paratype, specimen JAL14-9239, Figs 61, 62). Carapace length 2.3; abdomen length 2.4. Epigynum (Fig. 52) with central pocket parallel-sided, as typical for *clypeatus* and *viridipes* groups. Atrium small, crescent shaped, in front of a central pocket that has more or less parallel sides. Colour (Figs 61–62) pale except for dark patches on chelicerae in the same places as male. In alcohol there is a faint hint of the abdominal markings of the male.

#### Geographical variation.

Males from the coast of Jalisco, including the type locality and El Tuito, have brown faces (Fig. 58) and a third patella with a red-purple protuberance (Fig. 59). Males from north of the coastal mountain range, near El Grullo, have white hairs on the chelicerae and the border of the clypeus (Fig. 63), a third patella with no black spot and reduced protuberances that lack the red-purple colour (Fig. 68), and a first femur that is more obviously greenish (Fig. 63). Males from further north, in Nayarit, have an extensively white clypeus, a greenish first femur (Fig. 64), and a third patella with the black spot and protuberances that are black rather than red-purple. No difference was noted in the palpi, nor were striking differences noted in their courtship behaviours (see below, Natural History). It is possible that these forms represent separate species, but they are here retained together pending more data.

#### Additional material examined.

35♂♂ 7 ♀♀ in UBC-SEM: *Coastal (typical) form*: México: Jalisco: Chamela estuary, 19.5290°N 105.0770°W, 2 June 1998, W. Maddison et al., WPM#98-070 (5♂♂). México: Jalisco: north of El Tuito, 20.337°N 105.316°W, 3 June 1998, W. Maddison et al., WPM#98-072 (5♂♂ 1♀). México: Jalisco: Estación de Biología Chamela, Chachalaca Trail, 19.496°N 105.042°W, 10 Feb. 2014, W. Maddison & H. Proctor, WPM#14-015 (1♂). México: Jalisco: Estación de Biología Chamela, Viveros, 19.499°N 105.043°W, 90 m elev., 16 - 27 Feb. 2014, W. Maddison & H. Proctor, WPM#14-028 (1♂). México: Jalisco: Estación de Biología Chamela, 400-650 m on Calandria Trail, 19.5038 -19.5045°N 105.0334 - 105.0344°W, 19 Feb. 2014, W. Maddison & H. Proctor, WPM#14-034 (2♀♀). México: Jalisco: El Tuito, Rancho Primavera, 20.341°N 105.350°W, 600 m elev., 2 - 4 March 2014, W. Maddison & H. Proctor, WPM#14-044 (2♂♂). *El Grullo form*: México: Jalisco: Apulco, 19.737°N 103.903°W, 920 m elev., 31 May 1998, W. Maddison et al., WPM#98-064 (1♂). México: Jalisco: Los Yesos, near El Grullo, 19.750°N 104.067°W, 900 m elev., 1 June 1998, W. Maddison et al., WPM#98-065 (5♂♂ 1♀). México: Jalisco: Los Yesos, near El Grullo, 19.7508°N 104.0595°W, 870m elev., 1 June 1998, W. Maddison et al., WPM#98-066 (2♂♂ 1♀). *Nayarit form*: México: Nayarit: north of Compostela, 25 km S of Tepic, 21.323°N 104.921°W, 1000 m elev., oak woodland, 4 June 1998, W. Maddison et al., WPM#98-077 (5♂♂ 1♀). México: Nayarit: few km W of Compostela on highway 200, 21.2233°N 104.9382°W, 820 m, 4 June 1998, W. Maddison et al., WPM#98-078 (6♂♂ 1♀). México: Nayarit: few km W of Compostela on highway 200, 21.212°N 104.949°W, 870 m, 4 June 1998, W. Maddison et al., WPM#98-079 (2♂♂).

#### Natural history.

Collected in the tropical deciduous forests in Jalisco and Nayarit, México. It occurs on leaf litter (Fig. 100) along trails and small clearings (Fig. 101), exposed to the sun but with shade nearby. Courtship resembles that of the *viridipes* group, with an early stage in which the palps are waved in small circles and the first leg tips pointed at the female, followed by two transitional waves of the front leg, followed by a long period of asymmetrical flickers of the first legs. Coastal and Nayarit populations have similar displays (Coastal: ♂w259 https://youtu.be/rL24mLEkUxE, ♂w257 https://youtu.be/ty6p7NioFnU; Nayarit: ♂w261 https://youtu.be/i79aw5ju1EA, ♂w262 https://youtu.be/JV8AjMAgO58).

The pattern of light and dark spots or bands visible inside the male’s AME (Fig. 69) is an intriguing feature of *Habronattus roberti* and members of the *clypeatus* group. Maddison and Maddison (2016) discussed such visible patterns in the male eye as characteristic of the *clypeatus* group (Fig. 70; see also https://www.youtube.com/watch?v=Dq5ky7vjPYo). As one looks into the AME of most living salticids with translucent carapaces, one sees the colour changing from honey to black smoothly as the eye moves inside the prosoma and our line of sight moves from the side walls of the eye to the retina itself. The pattern of dark spots in *Habronattus roberti* and the *clypeatus* group is therefore unusual. It is unclear whether other *Habronattus* have such a pattern; the eye simply appears dark. In *Habronattus roberti*, as in the *clypeatus* group, the integument underlying the thoracic bands is unusually pale, which may permit light to enter the prosoma thus revealing the pattern. Given that this pattern is at the same focal plane as the ornamented third legs (Figs 69, 70), it is conceivable that the visibility of the eye pattern itself is a courtship ornament, enabled by the depigmented thorax.

### Habronattus sp. near carolinensis

Taxon classificationAnimaliaAraneaeSalticidae

(Peckham & Peckham, 1901)

Fig. 71

#### Note.

**Figure 71.**
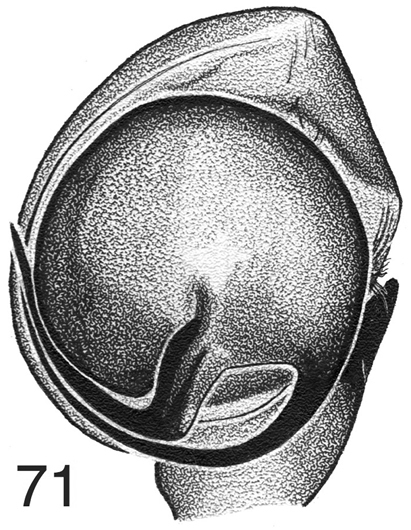
*Habronattus* cf. *carolinensis*, from Lake Temagami, Ontario, male left palp, ventral view.

At the Royal Ontario Museum in 1978 I saw a male specimen of *Habronattus* from Lake Temagami, Ontario, from whose label I recorded the collecting data “Ontario: Temagami. Island 1027. 24 June 1939. #5669”, although the museum reference notes indicated the date as 27 June 1937. It was notable for the brush of longer setae on the dorsal distal surface of the cymbium, and the twisted and tufted tarsus of the first leg. In both of these features it resembled the two described species *Habronattus carolinensis* (Peckham & Peckham, 1901) (from the southeastern U.S.) and *Habronattus venatoris* Griswold, 1987 (from the southern Rocky Mountains of Wyoming, Colorado, and New Mexico), both of which are notable for the twisted and tufted tarsus and metatarsus of the first leg (Chamberlin and Ivie 1944, figure 210). I drew the palp (Fig. 71), which differs distinctly in rotation of the bulb from those two species (embolus arising at 225°, compared to 270° for *Habronattus venatoris* and 290° for *Habronattus carolinensis*), and thus represents a new species. I did not draw the cymbial brush or the first leg, and my memory does not retain their details except that there was a clear resemblance to *Habronattus carolinensis* in these ornaments. Recent attempts to locate the specimen at the museum have failed, and it may have been loaned for a project on *Pellenes* (which was never completed) and not returned. In 1995 I travelled to the exact island in Lake Temagami on the label, but no specimens were found. However, the island was rock of perhaps 5 meters by 2 meters, unlikely to sustain any permanent population, and so either the specimen ballooned in, or the label was incorrect. We are thus left with a biogeographically puzzling new species with no specimen on which to describe it. Two possible habitats might be productively searched: the rock outcrops of the Canadian Shield, or exposed sand of glacial deposits in Northern Ontario and Québec.

### Genus Pellenes

Taxon classificationAnimaliaAraneaeSalticidae

Simon, 1876

#### Type species.

*Aranea tripunctata* Walckenaer, 1802

#### Notes.

Currently 84 species are assigned to *Pellenes* (World Spider Catalog, 2016). There are five subgenera: the nominate, three described by described by Logunov et al. (1999) and one by Prószyński (2016). These are:

*Pellenes* Simon 1876. Logunov et al. (1999) included three species in the nominate subgenus. The TmA is massive, much larger than the embolus. Logunov et al (1999) diagnosed *Pellenes* *s. str.* by “a raised epigynal median septum in females ... and the tibial apophysis appressed in the cymbial groove in males”.

*Pelpaucus* Logunov, Marusik & Rakov, 1999 (type species *Pellenes limbatus* Kulczyński, 1895). Six species are assigned to *Pelpaucus*. Their TmA is a more or less straight flat blade, wide especially at the tip, parallel to the embolus and as long as it. Logunov et al (1999) diagnosed *Pelpaucus* by the RTA very short or absent, an apical spine on the TmA, a recessed epigynal atrium, and a one-chambered spermatheca.

*Pelmultus* Logunov, Marusik & Rakov, 1999 (type species *Attus geniculatus* Simon, 1868). Twenty-three species are assigned to *Pelmultus*. They are compact-bodied (not elongate), with contrasting markings and a somewhat-ornamented male first leg, resembling in habitus to some extent the *Habronattus dorotheae* species group. The TmA is as long as the embolus but wider, with a complex pointed tip. Logunov et al (1999) diagnosed *Pelmultus* by the heavily sclerotized epigynal flaps and the subparallel tips of the embolus and the TmA.

*Pelmirus* Logunov, Marusik & Rakov, 1999 (type species *Pellenes dilutus* Logunov, 1995). Four species are assigned to *Pelmirus*, having a large complex TmA that curls distally at the tip, like a tongue. Logunov et al. (1999) diagnosed *Pelmirus* by the embolus and TmA perpendicularly orientated to each other and the peculiar elevated central pocket of the epigynum.

*Pellap* Prószyński, 2016 (type species implied to be *Pellenes lapponicus* Sundevall 1833). *Pellenes lapponicus* has a long embolus and TmA, the latter much wider. Prószyński (2016) characterizes *Pellap* by the long needle-like embolus sheathed in the thick TmA, trapezoidal RTA, medial groove behind the central pocket of the epigynum, and spiral spermathecae.

In all of these subgenera, the TmA is distinctly larger than the embolus. Many other species of *Pellenes* are not yet assigned to a subgenus, including many African species whose TmAs are small or (apparently) absent.

Although two Holarctic species are known from the Americas, Pellenes (Pellap) lapponicus and Pellenes (Pelpaucus) ignifrons (Grube, 1861), the remaining species of New World *Pellenes* form a distinctive group not known to occur in the Old World. The subgenus *Pellenattus* is here described to contain them.

### Pellenattus subgen. n.

Taxon classificationAnimaliaAraneaeSalticidae

http://zoobank.org/18078E5C-998D-4498-8D42-86536F0D6FF7

Figs 72–81 , 82–85 , 86–94

#### Type species.

*Pellenes peninsularis* Emerton, 1925

#### Diagnosis.

**Figures 72–81.**
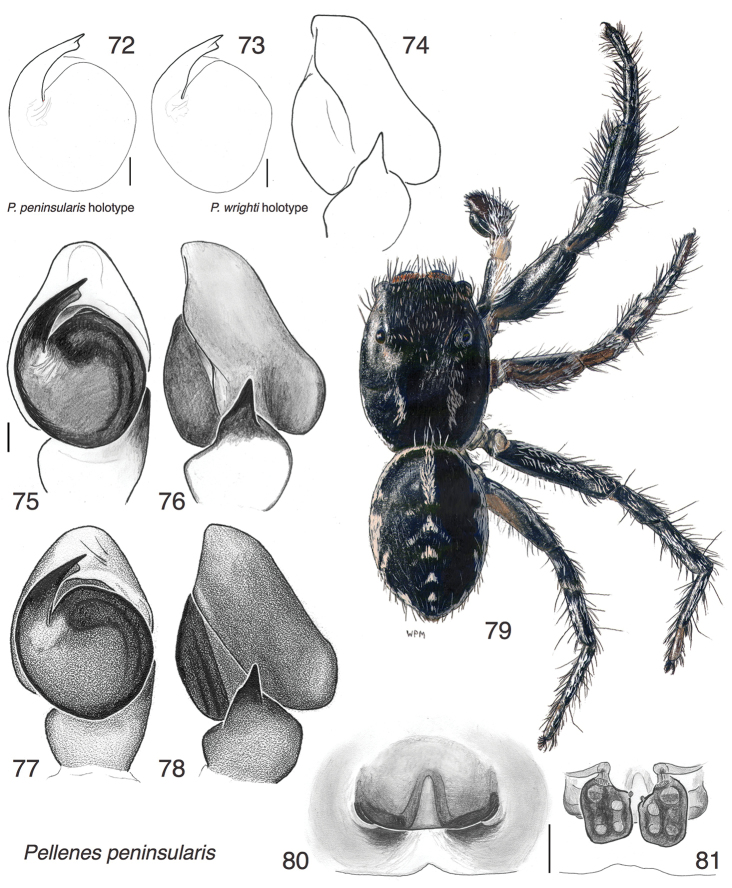
*Pellenes peninsularis*. **72** Bulb of left palp, holotype of *Pellenes peninsularis* Emerton, 1925 **73–74** Left palp, holotype of *Pellenes wrighti* Lowrie & Gertsch, 1955 **75–76** Left palp, male from Nova Scotia (DRM02.103) **77–78** Left palp, male from east-central Ontario (WPM#76-133) **79** Male from east-central Ontario (WPM#76-133) **80** Epigynum, ventral view, female from Nova Scotia (DRM02.103) **81** Epigynum, cleared, dorsal view, same female. DRM and WPM collecting codes are those indicated in specimen records.

**Figures 82–85.**
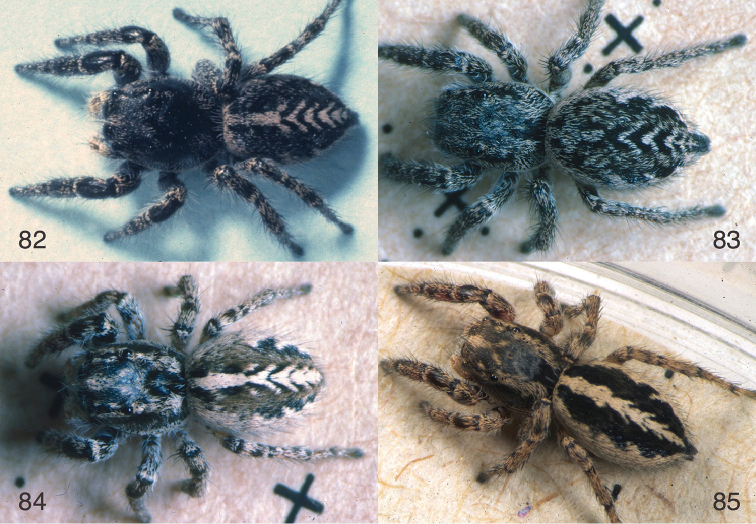
Female Pellenes (Pellenattus) species. **82** *Pellenes peninsularis* from Ontario: Muskoka District: Dwight **83** *Pellenes apacheus* from Arizona: Apache Co.: Mt. Baldy Wilderness, 33.92°N 109.63°W **84** *Pellenes limatus* Arizona: Pinal Co.: Three Buttes, 8.9 mi N of Oracle Junction along highway 79 **85** *Pellenes longimanus* from Texas: Hidalgo Co.: Bentsen-Rio Grande Valley State Park 26.178°N 98.391°W. Figs 82–85 are copyright © 2015 W. P. Maddison, released under a Creative Commons Attribution (CC-BY) 3.0 license.

**Figures 86–94.**
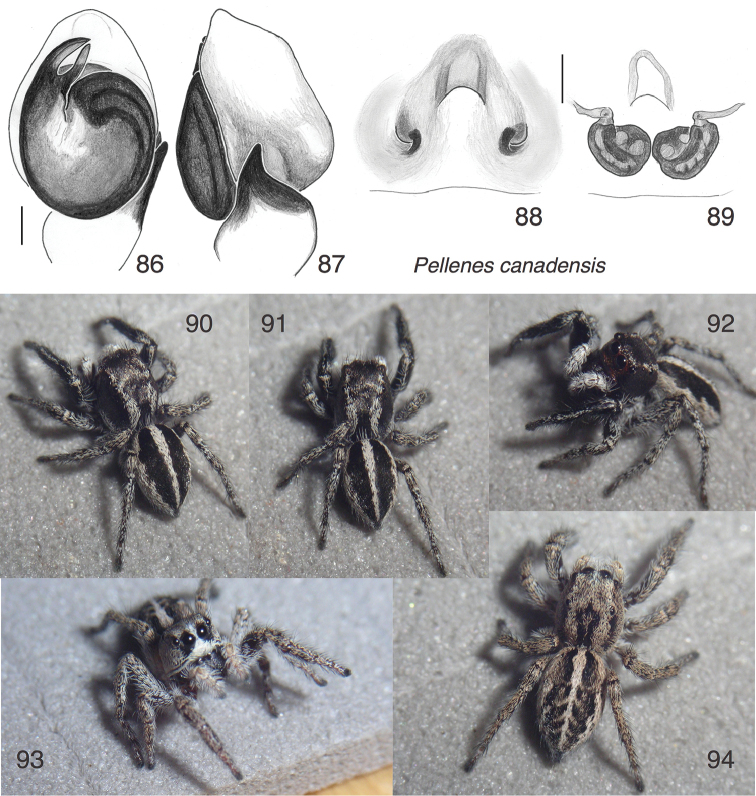
*Pellenes canadensis* sp. n. **86** Left male palp, ventral view (holotype male) **87** Same, retrolateral view of palp tibia **88** Epigynum, ventral view (paratype female NA13-6083) **89** Epigynum, cleared, dorsal view (same female) **90–92** Male paratype NA13-6071 **93–94** Female paratype NA13-6083 All specimens are from the type locality. Figs 90–94 are copyright © 2015 W. P. Maddison, released under a Creative Commons Attribution (CC-BY) 3.0 license.

Differs from the other described subgenera of *Pellenes* in having the TmA smaller than the embolus. The TmA of *Pellenattus* is often reduced to a small protuberance (Figs 72, 73, 75, 77, *Pellenes peninsularis*), or if as long as the embolus, then it is narrower than it (Fig. 86, *Pellenes canadensis*). In Old World species placed in described subgenera, the TmA is distinctly broader and larger than the embolus proper. The breadth of their TmA could be considered a synapomorphy of the four described subgenera, thereby excluding *Pellenattus*. Alternatively, the narrowness of the TmA in *Pellenattus* could be considered a synapomorphy with *Habronattus*. Those Old World species with a small TmA are primarily African (e.g., *Pellenes bulawayoensis* Wesołowska, 1999) and as yet unplaced to subgenus. *Pellenattus* species have a relatively narrow body and a simple medial longitudinal band, often divided into chevrons, on the abdomen (Figs 82–85), in contrast to *Pelmultus* and the African *Pellenes* which are more compact-bodied and have more contrasting markings. Strong transverse or oblique pale abdominal bands as seen in many Old World species (e.g., *Pellenes tripunctatus*, *Pellenes bulawayoensis*, *Pellenes nigociliatus* (Simon, 1875)) are absent from the American species. Molecular data currently being prepared for publication also support the distinctiveness of the American *Pellenes*.

The species here placed in *Pellenattus* are:

Pellenes (Pellenattus) apacheus Lowrie & Gertsch, 1955!, comb. n.

Pellenes (Pellenattus) canadensis sp. n.!

Pellenes (Pellenattus) crandalli Lowrie & Gertsch, 1955!, comb. n.

Pellenes (Pellenattus) dorsalis (Banks, 1898), comb. n.

Pellenes (Pellenattus) grammaticus Chamberlin 1925!, comb. n.

Pellenes (Pellenattus) levii Lowrie & Gertsch, 1955!, comb. n.

Pellenes (Pellenattus) limatus Peckham & Peckham, 1901!, comb. n.

Pellenes (Pellenattus) longimanus Emerton, 1913!, comb. n.

Pellenes (Pellenattus) peninsularis Emerton, 1925!, comb. n.

Pellenes (Pellenattus) shoshonensis Gertsch, 1934!, comb. n.

Pellenes (Pellenattus) washonus Lowrie & Gertsch, 1955!, comb. n.

Most of the described species of Pellenes (Pellenattus) were figured by Lowrie and Gertsch (1955). I have examined the holotypes of those species marked with ! in the list above. Although Griswold (1987) considered *Pellenes dorsalis* to be a *nomen dubium*, Banks’s figure of the palp almost certainly shows a *Pellenes* near *Pellenes washonus*, given the context of the Sonoran fauna. The figure of *Pellenes cinctipes* Banks, 1898 suggests it belongs here as well.

Logunov et al. (1999) placed one of these species within one of the Old World subgenera, *Pellenes limatus* into *Pelmultus*, but *Pellenes limatus* is a typical American species, differing from *Pelmultus* by the characters mentioned above.

### Pellenes (Pellenattus) peninsularis

Taxon classificationAnimaliaAraneaeSalticidae

(Emerton)

Figs 72–81 , 82

Pellenes peninsularis Emerton, 1925: p 68, fig. 6 (Dm) (male holotype in Museum of Comparative Zoology, Harvard, examined, Fig. 72)
Pellenes wrighti Lowrie & Gertsch, 1955: p. 23, fig. 19, 20, 27 (Dmf), **syn. n.**. (male holotype in American Museum of Natural History, examined, Figs 73–74)

#### Notes.

The vial of *Pellenes wrighti*’s holotype includes the labels “Pellenes wrighti Lowrie & Gertsch ♂ holotype”, “Pellenes peninsularis ♂, Ill., Kankakee Col, Pembroke TWP. SEC, Sept 8 1936, Coll. & Det. D.C. Lowrie”, and “Pellens [sic.] sp. nov. ♂ ♀ ♀ from Ind. Porter Co., Tremont. 8 June 1929”. The second (contradictory) locality label matches that expected for the female allotype of *Pellenes wrighti*, and may have been inserted or retained in error.

Lowrie and Gertsch (1955) compared *Pellenes wrighti* to *Pellenes apacheus* but made no comment about its differences from *Pellenes peninsularis*, despite the *Pellenes wrighti* holotype having been originally identified as *Pellenes peninsularis*. The holotypes of *Pellenes peninsularis* and *Pellenes wrighti* and other specimens from throughout the range bear no known features that would justify distinguishing two species. The bodies of specimens from Ontario and Nova Scotia are primarily black; those from the prairies of Minnesota, Montana and South Dakota are dusted with tan to orange scales, corresponding to their different substrates (dark rock outcrops in the eastern Canadian populations; prairies further west). There may be a small genitalic difference, in the size of the dorsal lobe of the cymbium, which appears to be larger in eastern than western populations (Figs 76, 78 versus Fig. 74). However, any difference would be slight, within the usual variability of a single species, and there is no indication of an abrupt transition in form in specimens arrayed from Nova Scotia through Montana. Otherwise, the male palp is consistent from west to east, with the TmA a small rounded flange pendant from the tip of the embolus (Figs 72, 73, 75, 77), the tibial apophysis leaning dorsally, and the cymbium with a prominent dorsal proximal lobe (Figs 74, 76, 78). It is possible that *Pellenes apacheus* is also conspecific, extending the trend to an even smaller cymbial lobe toward the southwest, but confirmation would require further study.

#### Additional material examined.

21♂♂ 19♀♀ in UBC-SEM: Canada: Nova Scotia: Pictou Co., Barneys River at route 104, 45.58617° N 62.22710° W, 22 June 2002, L.J. Maddison, D.R. Maddison, A.E. Arnold, DRM02.103 (1♂ 3♀♀). Ontario: Muskoka District, Dwight, 45.3384°N 79.0302°W, 22 May 1976, W. Maddison WPM#76-133 (1♂). Ontario: Muskoka District, Dwight, 45.3384°N 79.0302°W, 17 May 1980, W. Maddison, WPM#80-020 (15♂♂ 12♀♀). Ontario: Kenora District, Percy Lake, few mi E of Hawk Lake on HWY 17, 29 May 1977, W. & D. Maddison, WPM#77-122 (1♂ 1♀). U.S.A.: Minnesota: Anoka Co., Cedar Creek Natural History Area, Rg 23 W Twp 34 N Sect 34-35, 16 May 1977, D. & W. Maddison, WPM#77-025 (1♂ 2♀). North Dakota: Stark Co., S of Belfield, 74 km N of Bowman along HWY 85, 31 May 1982, D. Maddison, WPM#82-164 (1♂). South Dakota: Pennington Co., 12 mi W of Interior on HWY 44, 19 May 1977, W. Maddison, WPM#77-051 (1♂ 1♀).

#### Natural history.

This species occurs on rock outcrops in Nova Scotia and on the Canadian Shield of Ontario. Further west it occurs among grasses on the ground of prairies.

### Pellenes (Pellenattus) canadensis sp. n.

Taxon classificationAnimaliaAraneaeSalticidae

http://zoobank.org/1F25FF98-0859-41E1-A72F-F56771B28AD3

Figs 86–94

#### Holotype.

Male (Figs 86, 87) in UBC-SEM, with data: Canada: British Columbia: Mt. Baldy. 49.099°N 119.156°W, 1180 m elev. 17 May 2013 W.Maddison & H. Proctor WPM#13-014.

#### Paratypes

(3♂♂ 2♀♀). Same data as holotype (1♂ specimen NA13-6071 and 1♀ specimen NA13-6083 in UBC-SEM). Canada: British Columbia: W of Midway, along HWY 3, 3.0 km E of crossing of Kettle River with HWY 3, ca. 49.0°N 118.83°W, 2 May 1982, W. & D. Maddison, WPM#82-019 (1♂1♀ in CNC, 1♂ in AMNH).

#### Etymology.

Named for the country of the type locality, in honour of the 150th anniversary of Canada’s confederation.

#### Diagnosis.

A typical member of *Pellenattus* with striped markings, more contrasting in males than females. *Pellenes canadensis* can be distinguished by the TmA being only slightly smaller than the embolus, diverging from the embolus initially, then curving distally to touch the tip of the embolus. *Pellenes levii* has a similar palp, but its TmA is shorter and considerably narrower, only 1/4 to 1/3 the width of the embolus, and also is pressed against the embolus its entire length (Lowrie and Gertsch 1955, figure 17; holotype in AMNH examined).

#### Description.

*Male* (focal specimen: holotype). Carapace length 2.0; abdomen length 2.1. Structure of body typical for *Pellenattus*. Embolus a short pointed blade, accompanied by a TmA of almost the same size, which opposes the embolus like a thumb against a forefinger (Fig. 86). The RTA is broad but pointed (Fig. 87). The cymbial lobe is small but distinct, projecting toward the retrolateral (e.g., visible behind the RTA in Fig. 86). Colour: Black except for paler femur, patella and tibia of palp, and coxae and trochanters of legs. Body with longitudinal stripes of white scales (Figs 90–91).

*Female* (focal specimen: paratype, specimen NA13-6083, Figs 88, 89, 93, 94). Carapace length 2.4; abdomen length 2.7. Structure of body typical for *Pellenattus*. Central pocket not on a raised sclerotized mound (it is in *Pellenes peninsularis*). Openings posterior to central pocket (Fig. 86), as in *Pellenes levii* (Lowrie and Gertsch 1955, figure 29). Colour paler than male, with more distinct chevrons on the abdomen. Clypeus white except black patches below AME (Fig. 93).

#### Additional material examined.

U.S.A: Montana: Glacier Co., 1.3 mi SE of intersection of HWY U.S. 89, Cutbank River & HWY 445, 24 May 1977, D., W., L., & R. Maddison, WPM#77-099 (12 ♂♂ 7♀♀ 4 juveniles in UBC-SEM).

#### Natural history.

Collected at fairly high elevation on open ground with scattered small rocks, sticks and sparse vegetation (Fig. 98).

**Figures 95–101.**
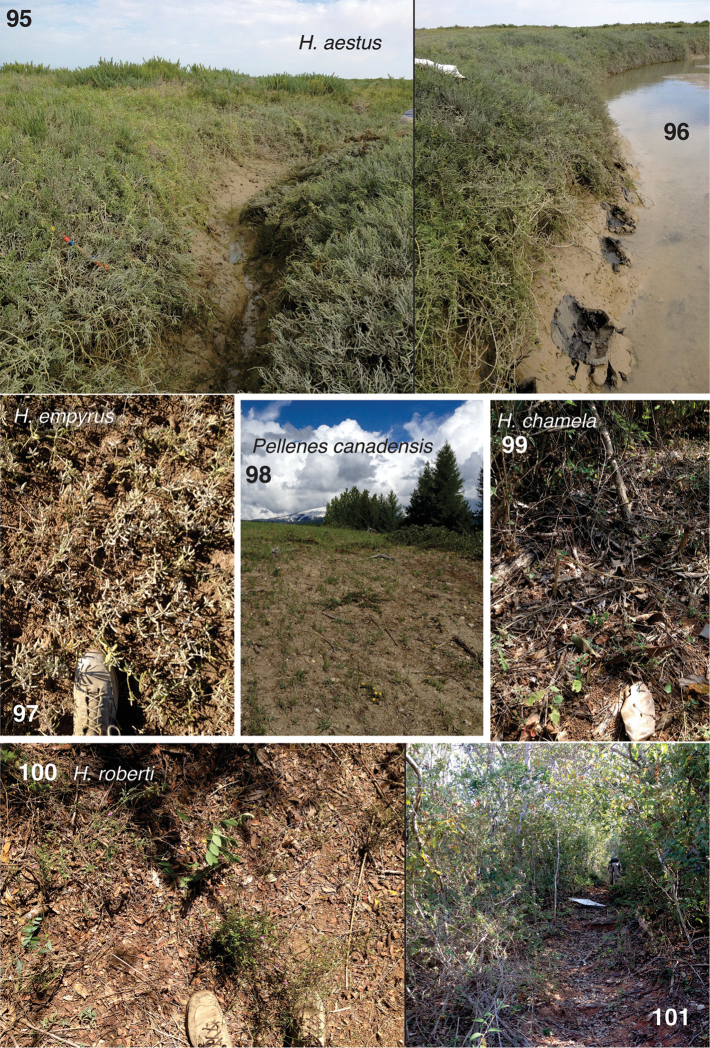
Habitats of new *Habronattus* and *Pellenes* species. **95–97** Negative estuaries near Puerto Peñasco, Sonora **95, 96** Habitat of *Habronattus aestus*, type locality, Estero Cerro Prieto **97** Habitat of *Habronattus empyrus*, type locality, Estero Morúa **98** Habitat of *Pellenes canadensis*, type locality, Mt. Baldy, British Columbia **99–101** Tropical deciduous forest of Chamela, Jalisco at type locality for both *Habronattus roberti* and *Habronattus chamela* **99** Habitat of *Habronattus chamela*, type locality **100** Habitat of *Habronattus roberti*, type locality.

**Figures 102–103.**
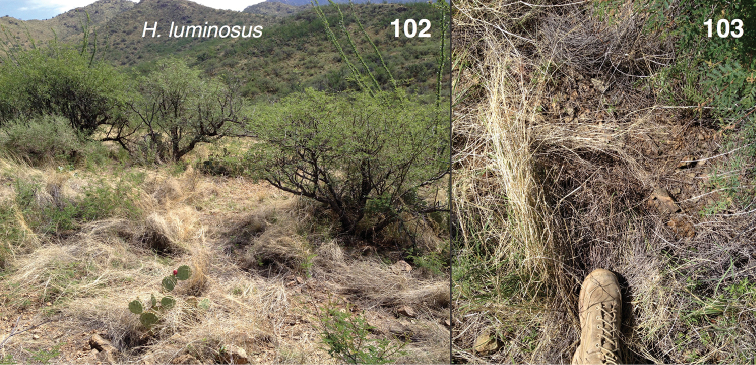
Habitat of *Habronattus luminosus*, Arizona, Amateur Astronomy Vista, 31.676°N 110.929°W.

## Supplementary Material

XML Treatment for Habronattus

XML Treatment for Habronattus aestus

XML Treatment for Habronattus chamela

XML Treatment for Habronattus empyrus

XML Treatment for Habronattus luminosus

XML Treatment for Habronattus roberti

XML Treatment for Habronattus sp. near carolinensis

XML Treatment for Pellenes

XML Treatment for Pellenattus

XML Treatment for Pellenes (Pellenattus) peninsularis

XML Treatment for Pellenes (Pellenattus) canadensis
